# Opinion formation with time-varying bounded confidence

**DOI:** 10.1371/journal.pone.0172982

**Published:** 2017-03-06

**Authors:** YunHong Zhang, QiPeng Liu, SiYing Zhang

**Affiliations:** 1 Institute of Complexity Science, Qingdao University, Qingdao, China; 2 College of Computer Science & Technology, Qingdao University, Qingdao, China; Peking University, CHINA

## Abstract

When individuals in social groups communicate with one another and are under the influence of neighbors’ opinions, they typically revise their own opinions to adapt to such peer opinions. The individual threshold of bounded confidence will thus be affected by both a change in individual confidence and by neighbor influence. Individuals thus update their own opinions with new bounded confidence, while their updated opinions also influence their neighbors’ opinions. Based on this reasoned factual assumption, we propose an opinion dynamics model with time-varying bounded confidence. A directed network is formed by the rule of the individual bounded confidence threshold. The threshold of individual bounded confidence involves both confidence variation and the in/out degree of the individual node. When the confidence variation is greater, an individual’s confidence in persisting in his own opinion in interactions is weaker, and the individual is more likely to adopt neighbors’ opinions. In networks, the in/out degree is determined by individual neighbors. Our main research involves the process of opinion evolution and the basic laws of opinion cluster formation. Group opinions converge exponentially to consensus with stable neighbors. An individual opinion evolution is determined by the average neighbor opinion effect strength. We also explore the conditions involved in forming a stable neighbor relationship and the influence of the confidence variation in the convergence of the threshold of bounded confidence. The results show that the influence on opinion evolution is greater with increased confidence variation.

## Introduction

People often communicate their daily concerns with others. When an individual’s opinion differs from that of others, he is likely to heuristically revise his opinion to adapt to his peer’s behavior, with some changes in his confidence [1,2]. In social behavior, individual opinion interactions depend on various social or psychological factors, such as personality, trust level, reputation, social status, and persuasive abilities [3,4]. Research has shown that opinion and confidence are related at the outset; when most of an individual’s peers have similar opinions, that individual may choose to compromise his opinion [3]. However, when those same peers have opinions that are similar to the individual’s opinions, it can boost an individual’s confidence–even to the level of a related effect known as overconfidence [5, 6]. In the latter context, an individual with strong self-confidence will doubt the accuracy of his own judgment and will be willing to adjust his confidence level to adapt to the collective opinion [7]. In such a context, to confirm that a person’s opinion is similar to another person’s opinion is more important in social behaviors than to express the correctness of the individual opinion [8, 9].

Opinion dynamics theory analyzes how individuals choose conventions, make decisions, schedule tasks, and implement actions [10]. At a certain scale of social groups, the rules concerning the local interactions among members may rise to complex behavior based on opinion dynamics, such as consensus, polarization and fragmentation [11–13]. In recent years, statistical physics has been widely applied to the social sciences, and an opinion dynamics model with the characteristics of statistical physics has been developed in social opinion research, which can reveal the emergence of collective behavior characteristics in local individual interaction rules. Based on different assessments of the value of opinion, the previous literature mainly focuses on discrete and continuing opinion dynamic models. The discrete models include the Ising spin model [14], the voter model [15], the majority rule model [16], the social impact model [17], and the Sznajd model [18], whereas the continuing models mainly refer to the Deffuant model [19], and the HK model [20]. A comprehensive survey of these models can be found in the references [21,22].

The opinion dynamics model is also helpful in exploring the formation of opinion clusters and reveals the emergence of collective interaction behaviors in networks [23]. The previous literature [24] analyzes the formation of opinion clusters in adaptive networks, while the related literature [25] examines how community structure affects opinion clusters. In the previous literature [26], research explores the influence of short-and long-range interactions with individual bias on the overall meaning of opinion clusters.

In the current research on opinion dynamics, individuals are typically assumed to be homogenous and to share the same bounded confidence level, such as the assumptions found in the DW and HK models; however, individuals are different both physiologically and psychologically, and they often have differential reactions in the face of uniform peer opinion. Moreover, individuals may make different decisions with biased confidence levels. Thus, assessing individuals in models as heterogeneous is generally reasonable. A heterogeneous DW model and an HK model were explored in [27] as interactive Markov chains and in [28] as opinion evolutions that included an agent-based version and a density-based version. Heterogeneous individuals have been segregated based on their confidence levels into informed and uninformed [29] or multi-levels [10],whereas a heterogeneous model with a time-variant interaction topology was analyzed in [30,31],and a consensus study was explored in an agent-based model with a time-varying directed graph in [32].These opinion dynamics models are becoming increasingly close to real social interaction behaviors, as they incorporate heterogeneous individuals, changeable confidence levels and time-varying interaction topologies.

To survey the characteristics of opinion dynamic behavior in social behaviors, we propose a model with nodes that are related to individual confidence level and an in/out degree of neighbor node. The model is an agent-based and based on a continued opinion dynamics model [19,20]in which the opinion of an agent can vary smoothly between extremes – in contrast to discrete opinions, such as binary values, with inherent yes or no responses [15,16,18] – with a bounded confidence rule that stipulates that agents can interact with one another when the distance of their opinions is close enough to a given confidence level[19,20,22]. In addition, in this model individuals have different initial thresholds of bounded confidence – which is a critical value of interaction between agents who are willing to exchange opinions – and a confidence level described by a variable that can change overtime, further an agent can revise his confidence level in his interactions with others, this heterogeneous confidence is called ‘confidence variation’. The agent determines his new threshold of bounded confidence based on this confidence variation and the in/out degree of neighbor nodes; his new threshold of bounded confidence will also impact his neighbor’s opinions in subsequent interactions. Thus, the threshold of bounded confidence is time-varying. The agent’s opinion undergoes a unidirectional change, i.e., the agent will revise his opinion to become closer to his peer’s opinion when his threshold of bounded confidence is less than his peer’s threshold; however, his peer will remain unaffected unless his peer’s own threshold is less than the agent’s threshold. The lower confidence level makes the individual insist on his opinion; therefore, his peers’ opinions will affect him less [3]. In this paper, we investigate the process of opinion evolution and the basic law of opinion formation.

The remainder of this paper is organized as follows. We begin with a definition of our model in Section 2. An analysis of opinion cluster formation and evolution is described in Section 3 and is followed by the experimental design and simulation results in Section 4.A conclusion is presented in the last section.

## Methods

### The time-varying bounded confidence model

In the Deffuant model [8], simple opinion dynamics were proposed for the fully connected graph in which all nodes were interconnected. Here, we apply the idea of neighbors connected by bidirectional or unidirectional links. A directed network G(t) = (V(t),(E(t)), V(t) consists of the nodes set at time t, and E(t) indicates the edges set at time t. In i,j ∈ V(t), node(agent) j is the neighbor of node i only if there is a link from j to i. Therefore $a_{ij}^{t}=1$, $a_{ij}^{t}\in A^{+}(t)$ and A^+^(t) is the in-degree adjacent matrix of G(t); otherwise, $a_{ij}^{t}=0$. Meanwhile $b_{ji}^{t}=1$, $b_{ji}^{t}\in A^{-}(t)$, and A^−^(t) is the out-degree adjacent matrix of G(t); otherwise, $b_{ji}^{t}=0$. The agents in a network have an opinion space that is denoted by X = [0,1] for some social issues, and the opinion of Agenti at time t is x_i_(t) ∈ X. The bounded confidence rule for Agentsi and j is ‖x_i_(t) − x_j_(t)‖ ≤ ε_i_(t), and the function of ε_i_(t) is:

$$\varepsilon_{i}(t)=(1-\delta (t))\varepsilon_{i}(0)+\delta (t)\frac{k_{i}^{+}(t-1)}{k_{i}^{+}(t-1)+k_{i}^{-}(t-1)}$$ (1)

When t = 0, ε_i_(t) = ε_i_(0) and ε_i_(0) is the initial threshold value of bounded confidence – which is a critical value that indicates how susceptible an agent is to the influence of the opinion of other agents – each agent has his own value at the beginning, and the confidence level for each agent is a variable in the [0.0,1.0]range with an initial value of 1. δ(t) = min{δ(t − 1) + Δδ * t, 1} is the confidence variation, and Δδ is the confidence variation increment, 0 < Δδ < 1. $\frac{k_{i}^{+}(t-1)}{k_{i}^{+}(t-1)+k_{i}^{-}(t-1)}$ is related by the in/out degree of Agenti and is used to express the influence of neighbors on Agenti's opinion, and t > 0. If the in-degree $k_{i}^{+}(t-1)=\sum_{j}\;a_{ij}^{t-1}$ is larger than the out-degree $k_{i}^{-}(t-1)=\sum_{j}\;b_{ij}^{t-1}$, thenthe confidence level of Agenti is relatively high, and the agent’s opinion depends much more on his neighbors. By contrast, when $k_{i}^{-}(t-1)$ is greater than $k_{i}^{+}(t-1)$, Agenti has a lower confidence level. If $k_{i}^{-}(t-1)$ and $k_{i}^{+}(t-1)$ are balanced, Agenti is open, and his confidence is not easily changed; thus the influence of his neighbors is not essential to his opinion. In this instance, the threshold of bounded confidence is time-varying because the agent changes his confidence following exposure to neighbors’ opinions.

After each round of opinion interactions, the agent will revise his opinion using certain rules to update his neighbors based on his confidence level, and he will weigh the opinions of his neighbors, on average, against his own opinion at the next interaction [3, 10,20]. However, we believe that it is more reasonable to consider an individual as a rational agent who can stick to his opinion at first and is thereafter influenced by his peers, such that the more closed the relationships are, the more influence they have in the evolution of the individual’s opinion. The rules of opinion evolution for each agent are provided in formula (2).The opinion of Agenti at the t time step is

$$x_{i}(t)=x_{i}(t-1)+\frac{1}{M_{i}^{t-1}}\sum_{j\in N_{i}(t-1)}a_{ij}^{t-1}\omega_{ij}[x_{j}(t-1)-x_{i}(t-1)]$$ (2)

where ω_ij_ ∈ W is the weighted relationship between Agentj and Agenti; the weighted spaceis W = [0,0.5], which indicates that the opinion of each agent will be affected half as strongly by his neighbors for the most part; and ω_ij_[x_j_(t − 1) − x_i_(t − 1)] is the effect strength of neighborAgent j to Agenti. $M_{i}^{t-1}$ is the number of neighbors for Agenti at the t − 1 time step. At the t time step, Agent i’s opinion depends on his own opinion and the average effect strength of his neighbor’s opinion at the t-1time step.

### Neighbor relationship for opinion formation

The neighbor relationship is important to opinion formation. For example, in a seminar in which participants exchange opinions with one another and reach an agreement, this neighbor relationship is bidirectional. In the case of media information regarding social issues that influences people’s opinions, the neighbor relationship is unidirectional. In a political election, different parties have different political opinions, and most of these opinions are in opposition to one another. To explore the influence of the neighbor relationship on opinion formation with time-varying bounded confidence, a definition of the neighbor relationship is given for Agentsi and j in G(t) as follows.

1. Bidirectional neighbors: For Agenti and Agentj, if ‖x_i_(t) – x_j_(t)‖ ≤ ε_i_(t) ≤ ε_j_(t) or ‖x_i_(t) – x_j_(t)‖ ≤ ε_j_(t) ≤ ε_i_(t), Agenti and Agentj are bidirectional neighbors of one another. All bidirectional neighbors are denoted as N_↔_(t) = {i,j ∈ V(t): (i,j) ∈ E(t),(j,i) ∈ E(t)}. All nodes in N_↔_(t) are connected fully and form a strongly connected network.
2. Unidirectional neighbors: For Agenti and Agentj, if ε_j_(t) ≤ ‖x_i_(t) – x_j_(t)‖ ≤ ε_i_(t), Agentj is the neighbor of Agenti, but Agenti is not the neighbor of Agentj. All unidirectional neighbors are denoted as N_←_(t) = {i,j ∈ V(t): (i,j) ∈ E(t),(j,i) ∉ E(t)}.
3. Non-neighbors: For Agenti and Agentj, if ‖x_i_(t) – x_j_(t)‖ > ε_i_(t) or ‖x_i_(t) – x_j_(t)‖ > ε_j_(t), Agentsi and Agentj are non-neighbors of one another. All the non-neighbors are denoted by N_<>_(t) = {i,j ∈ V(t): (i,j) ∉ E(t),(j,i) ∉ E(t)}.
4. Stable neighbor relationship: At the t time step, Agenti and Agentj are neighbors ofone another whose relationship is defined by one of the above definitions. If at the t + n time step, the relationship between Agenti and Agent j has not changed and continues for any t + n + l time step, and n ≥ 0,l > 1 then this is a stable neighbor relationship.

As the neighbor relationship plays an important role in opinion evolution, the law of opinion evolution must be further explicated.

## Discussion

In the HK model, the number of the final opinion cluster depends on the homogeneous confidence level [20,22]. However, what are the main factors in our model regarding opinion evolution? What is the influence of the neighbor relationship in opinion formation?

### Impacts of neighbor relationship

When individuals land in a new human social environment, they will most likely communicate with others who have similar characteristics and interests. As exchanges deepen, they form stable relationship networks.

If there is a stable neighbor relationship at the t + n time step, the opinion of Agenti at time t + n + 1 is redefined based on formula (2) as follows:

$$x_{i}(t+n+1)=x_{i}(t+n)+\frac{1}{M}\sum_{j\in N}\omega_{ij}[x_{j}(t+n)-x_{i}(t+n)]$$ (3)

where M is the number of neighbors and N is the set of neighbors of Agenti. Because of the stable neighbor relationship, M and N do not change with time.

When opinion evolution occurs at the t + n time step and there is a stable bidirectional neighbor relationship, we use Theorem 1.

***Theorem 1*:** *At the t* + *n time step*, *N*_↔_(*t*) *is set for any Agenti and Agentj who are bidirectional neighbors; then*, *at the t* + *n* + *l time step*, *the difference of their opinion value is* (*1 − Θ*)^*l*^ *times in the t* + *n time step, and* $\Theta =\frac{1}{M}\sum_{p,q\in N}\omega_{pq}$*, M represents the agents in N*_*↔*_(*t*).

Based on formula (3), we deduce the differences in opinion for Agenti and Agent j (who are bidirectional neighbors) as follows:

$$\begin{array}{l} x_{j}(t+n+1)-x_{i}(t+n+1)\\ \;=x_{j}(t+n)-x_{i}(t+n)+\frac{1}{M}[\sum_{q\in N}\omega_{jq}x_{q}(t+n)-\sum_{p\in N}\omega_{ip}x_{p}(t+n)]\\ \;-\frac{1}{M}[\sum_{q\in N}\omega_{jq}x_{j}(t+n)-\sum_{p\in N}\omega_{ip}x_{i}(t+n)]\\ \;=x_{j}(t+n)-x_{i}(t+n)\\ \;-\frac{1}{M}[x_{j}(t+n)-x_{i}(t+n)]\left(\omega_{ji}+\omega_{ij}+\sum_{p,q\in N-\{i,j\}}\omega_{pq}\right)\\ \;=\left[x_{j}\left(t+n\right)-x_{i}\left(t+n\right)\right]\left(1-\frac{1}{M}\sum_{p,q\in N}\omega_{\mathrm{pq}}\right)\\ \;=\left[x_{j}\left(t+n\right)-x_{i}\left(t+n\right)\right]\left(1-\Theta \right) \end{array}$$

Additionally, x_j_(t + n + l) − x_i_(t + n + l) = [x_j_(t + n) − x_i_(t + n)](1 − Θ)^l^, and ω_ij_ ∈ [0,0.5]; therefore, Θ ∈ [0,0.5], then ‖x_j_(t + n + l) − x_i_(t + n + l)‖ ≤ ‖x_j_(t + n) − x_i_(t + n)‖ ≤ ε_j_(t + n) or ‖x_j_(t + n + l) − x_i_(t + n + l)‖ ≤ ‖x_j_(t + n) − x_i_(t + n)‖ ≤ ε_i_(t + n). Thus, at the t + n + l time step, Agentsi and j are also bidirectional neighbors, and the difference of their opinion value is (1 − Θ)^l^ times in the t + n time step.

***Definition 1*:** *If neighbor sets of* $N_{↔}^{1}(t)$ *and* $N_{↔}^{2}(t)$ *exist in which the agents have bidirectional neighbor relationships, then for all* ${i\in G}_{↔}^{1}(t)$ *and* ${j\in G}_{↔}^{2}(t)$*, there is* (*i*,*j*) ∉ *E*(*t*) *and* $\left(j,i)\notin E(t)N_{↔}^{1}(t\right)$*, and* $N_{↔}^{2}(t)$ *have mutual independence, and* $N_{↔}^{1}(t)\cap N_{↔}^{2}(t)=\emptyset$.

***Theorem 2*:** *At time t, m sets exist that are stable with mutual independence,* $N_{↔}^{k}(t)=(V^{k}(t),E^{k}(t))$*, k = 1,2,…,m. A unidirectional directed network can be described as N*_*←*_(*t*) = {*i* ∈ *V*^1^(*t*), *j* ∈ *V*^*m−*1^(*t*): (*i*,*j*) ∈ *E*(*t*),(*j*,*i*) ∉ *E*(*t*)}, $V^{m-1}(t)={⋃}_{l=2}^{m}V^{l}$. *The evolutionary trend of an opinion cluster formed by the nodes’ opinions belongs to V*^*l*^(*t*) *in N*_←_(*t*) *and is related to the overall effect strength of neighbors’ opinions S*_*t*_. *If S*_*t*_ > *0*, *it will move ahead to a higher interval; if S*_*t*_ *< 0*, *it will move ahead to a lower interval; and if S*_*t*_ *= 0*, *it moves ahead*. *Moreover*, *if there is sufficient time*, *the opinion cluster will converge to consensus*.

Based on the formula (3), there is:

$$\begin{array}{l} x_{i}\left(t+n+1\right)-x_{i}\left(t+n\right)\\ \;=\frac{1}{M_{N_{\leftarrow }}}[\sum_{j\in N_{\leftarrow }\left(t+n\right)}a_{ij}^{t+n}\omega_{ij}\left[x_{j}\left(t+n\right)-x_{i}\left(t+n\right)\right]\\ \;=\frac{1}{M_{N_{\leftarrow }}}\{\sum_{j\in V^{1}\left(t\right)}\omega_{ij}\left[x_{j}\left(t+n\right)-x_{i}\left(t+n\right)\right]\\ \;+\sum_{j\in V^{2}\left(t\right)}\omega_{ij}\left[x_{j}\left(t+n\right)-x_{i}\left(t+n\right)\right]+\cdots \\ \;+\sum_{j\in V^{m}\left(t\right)}\omega_{ij}\left[x_{j}\left(t+n\right)-x_{i}\left(t+n\right)\right]\}\\ \;=\frac{1}{M_{N_{\leftarrow }}}\sum_{k=1}^{m}\{\sum_{j\in V^{k}\left(t\right)}\omega_{ij}\left[x_{j}\left(t\right)-x_{i}\left(t\right)\right]{\left(1-\Theta^{k}\right)}^{n}\}=\frac{1}{M_{N_{\leftarrow }}}\sum_{k=1}^{m}S_{i,t}^{k}{\left(1-\Theta^{k}\right)}^{n} \end{array}$$

and $\Theta^{k}=\frac{1}{M^{k}}\sum_{p,q\in V^{k}(t)}\omega_{pq}$, $S_{i,t}^{k}=\sum_{j\in V^{k}(t)}\omega_{ij}[x_{j}(t)-x_{i}(t)]$.

There is $\Delta X=\sum_{i\in V^{l}}[x_{i}(t+n+l)-x_{i}(t+n)]=\frac{1}{M_{N_{\leftarrow }}}\sum_{i\in V^{l}}\sum_{k=1}^{m}{S_{i,t}^{k}(1-\Theta^{k})}^{n}$ and $S_{t}=\sum_{i\in V^{l}}\sum_{k=1}^{m}S_{i,t}^{k}$ because (1 − Θ^k^) > 0, and the trend of the opinion cluster consisting of V^1^(t) in N_←_(t) is related to S_t_; lim_n→∞_ X = 0 when n is long enough, and the opinion in N_←_(t) will be the same and a consensus will be reached.

### Analysis of Δδ forthe time-varying threshold of bounded confidence

Individuals have different confidence levels and will thus choose to trust some individuals and distrust others; as the trusted persons are grouped together to form a group, they will have similar confidence levels.

For all agents, there is an Agenti whose trust threshold value is ε_i_(t + n) at the t + n time step. Based on formula (1), we have

$$\varepsilon_{i}(t+n)=(1-\delta (t+n))\varepsilon_{i}(0)+\delta (t+n)\frac{k_{i}^{+}(t+n-1)}{k_{i}^{+}(t+n-1)+k_{i}^{-}(t+n-1)}$$ (4)

***Theorem 3***: *At time t*, *a set N*(*t*) *with M nodes and i* ∈ *N*(*t*) *exists*, *and the confidence variation increment of Agenti is 0* < Δ*δ* ≤ 1; *at time t + n*, *Agenti and his neighbor have a stable neighbor relationship*. *The in and out degrees are k*^*+*^ *and k*^*−*^ *for all nodes in set N*(*t*)*, such that* $K=\frac{k^{+}}{k^{+}+k^{-}}$*. When K < ε*_*i*_(0), *the time-varying threshold of bounded confidence for Agenti is decreased progressively at* δΔε_i_. *When K > ε*_*i*_(0), *the time-varying threshold is increased progressively at* δΔε_i_. *When K = ε*_*i*_(0), *the time-varying threshold does not change overtime*. *Thus*, *at this juncture*, Δε_i_ *= K − ε*_*i*_(0)*. When* $K=\frac{1}{M}\sum_{i}\varepsilon_{i}(0)$*, the time-varying bounded confidence converges to consensus.*

For any agent at the t + n + 1 time step, we can infer as follows based on formula (4):

$$\begin{array}{l} {[\varepsilon }_{i}(t+n+1)-\varepsilon_{i}(t+n)]\\ \;=[\delta (t+n)-\delta (t+n+1)]\varepsilon_{i}(0)+\delta (t+n+1)K-\delta (t+n)K\\ \;=\Delta \delta [K-\varepsilon_{i}(0)] \end{array}$$

If 0 < Δ*δ* ≤ 1, the change trend of [ε_i_(t + n + 1) − ε_i_(t + n)] depends on Δ*ε*_*i*_. Additionally, the total difference of time-varying bounded confidence is

$$\begin{array}{c} \sum_{i}{[\varepsilon }_{i}(t+n+1)-\varepsilon_{i}(t+n)]=-\Delta \delta \sum_{i}\left[\varepsilon_{i}(0)-K_{i}(t+n)\right]\\ =-\Delta \delta [\sum_{i}\varepsilon_{i}(0)-MK] \end{array}$$

Therefore, if $K=\frac{1}{M}\sum_{i}\varepsilon_{i}(0)$, the time-varying bounded confidence converges.

## Results

Experimental hypotheses on the initial conditions are provided to better investigate the dynamic behavior of an opinion cluster in simulation and instanceanalysis.

### Experimental hypothesis

1. There are200 agents in total, and each is coded for identification purposes from 1 to 200.
2. An agent’s opinion and his thresholds of bounded confidence obey a uniform distribution of [0,1.0], where the weight factor ω satisfies the uniform distribution of [0, 0.5].
3. There is no isolated node in the network, and all agents are willing to exchange opinions.
4. The confidence variation increment is given by the four conditions (Δδ = 0, Δδ = 0.005, Δδ = 0.01, Δδ = 0.9), that are described as Conditions I-IV in the following sections.
5. An opinion evolution period is called a time step in each experiment.

Next, we will reveal how opinion clusters are formed with experimental methods.

### Experiment result

**Fig 1:**
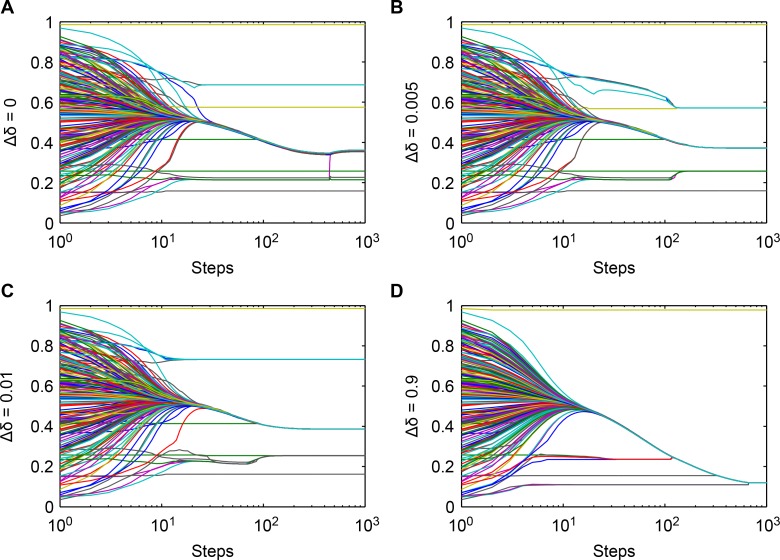
Opinion evolution with different values of Δδ. (**a**) Δδ = 0; (**b**) Δδ = 0.005; (**c**) Δδ = 0.01; (**d**) Δδ = 0,9. The lines with different color denote the opinions of agents varying from 0.0 to 1.0.

Opinion evolution is simulated in SWARM programming [33]. Fig 1 shows the evolution of opinions, and the confidence variation plays a prominent role under the four conditions, which is shown in the figures.

Fig 1 shows the opinion evolution over 1,000 time steps in Conditions I-IV. The times differ for opinion cluster formation, and the interval distribution of the largest opinion cluster changes from high to low due to different confidence variations. Condition IV changes dramatically.

The largest opinion clusters in the four conditions change downwards under the influence ofthe effect strength of neighbors’ opinions. To investigate the influence of the neighbor relationship on the agent’s opinion, we choose Agent117(the agent identified as 117), who has been observed in the biggest opinion cluster in Conditions I-IV in the experiments.

**Fig 2:**
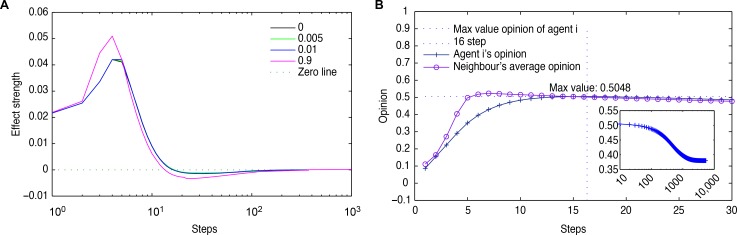
The opinion evolution of agent117. (a) The average effect strength of the opinions of Agent117’s neighbors. The black, green, blue, and red lines denote the confidence variations with Δδ = 0, Δδ = 0.005, Δδ = 0.01, and Δδ = 0.9, respectively. (b) Opinion evolution of Agent117 with Δδ = 0.01. The line with cross marks denotes the opinion of Agent117, and the line with circle marks denotes the average opinions of Agent117’s neighbors. The cross point of the horizontal and the vertical dotted lines indicate the max value of opinions for Agent117 and his neighbors. The embedded figure shows the opinion evolution of Agent117 in 10000 time steps.

We analyze the effect strength of his neighbors’ opinions in Conditions I-IV, which is shown in Fig 2A; all the lines in the figure first increase sharply, then decrease to less than zero, and finally increase slowly until they are steady at zero. The special situation is studied in Fig 2B for Condition III, where the neighbor relationship of Agent 117 is steady at the seventh time step, which is known from his in-degree and out-degree adjacent matrixes. However, at the 16th time step, his opinion changes to the max value, meeting in the average neighbor opinion value, and the effect strength of his neighbors’ opinions is more than zero at this time step. Then, his opinion decreases gradually along with his neighbors’ opinions effect strength to less than zero and converges over 10,000 time steps.

We find that there are different neighbor relationship sets in-degree and out-degree adjacent matrixes, such as bidirectional neighbors sets $N_{i↔j}^{7}=\{19,46,72,103,140,191,196\}$, $N_{i↔j}^{7}=\{29,42,109,144\}$, $N_{i↔j}^{1}=\{35\}$, and $N_{i↔j}^{186}=V-N_{i↔j}^{7}-N_{i↔j}^{4}-N_{i↔j}^{1}-N_{i↔j}^{6}$; the non-neighbors set $N_{i↔j}^{6}=\{6\}$; and one unidirectional neighbors set $N_{i↔j}^{186+}=\{j\in N_{i↔j}^{7}\cup N_{i↔j}^{4}\cup N_{i↔j}^{1},\;i\in N_{i↔j}^{186}:(i,j)\in E,(j,i)\notin E\}$; The sign V represents the set of all nodes, the superscript of the set N is the label (which is referred to here as the number of nodes), and the subscript is the relationship between agents. The numbers in sets serve to identify the agents. And we have got the sum of effect strength of opinions of agent 117’s neighbours in 1000 time steps from the data source as follows, and please see data file [S1 Dataset] for the specific calculation:

$$S_{1000}=S_{117,1000}^{7}+S_{117,1000}^{4}+S_{117,1000}^{1}+S_{117,1000}^{186}=-0.001036445$$

The opinion values of agents in set $N_{i↔j}^{186}$ change less because their neighbors with small opinion values have a greater effect regarding the strength of their opinions, such that the largest opinion cluster changes downwards.

Based on Theorem 2, we confirm that a group of random data must exist regarding opinions as well as a threshold and weight factor for those agents that make the opinion cluster move ahead to a higher level. Therefore, we simulate them in SWARM, and the results are as follows.

**Fig 3:**
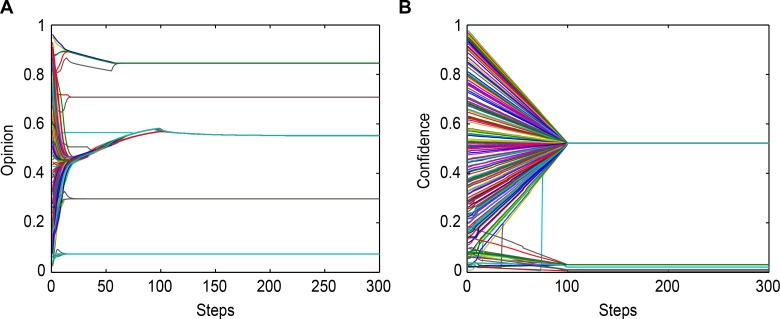
Opinion and confidence evolution with Δδ = 0.01. (a) Opinion Evolution. (b) Confidence Evolution. Lines with different colors denote the opinions and thresholds of bounded confidence.

Fig 3 shows that the largest opinion cluster moves ahead to a higher level with the influence of neighbor effect strength on other random data for the opinion of agents.

**Fig 4:**
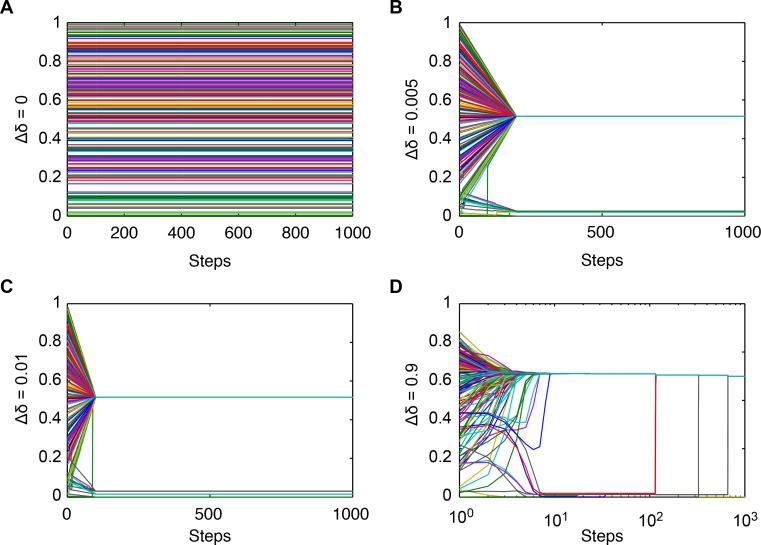
The threshold bounds of confidence with different values of Δδ. (**a**) Δδ = 0; (**b**) Δδ = 0.005; (**c**) Δδ = 0.01; (**d**) Δδ = 0.9. The lines with different colors denote the confidence level of agents varying from 0.0 to 1.0.

Fig 4 displays the changes in the threshold of bounded confidence in Conditions I-IV, In Conditions II-IV, the confidence level converges in different groups, and the convergent value is the average value of the group, i.e., when they have the same threshold of bounded confidence, they have a stable relationship and are in the same group, which is consistent with the results and theoretical analysis embodied in Theorem 3.

Fig 4 presents the thresholds of bounded confidence changes in the four conditions; in Condition IV, the best cluster of the trust threshold value is larger than the others.

When the confidence variation is larger, we find that the largest opinion cluster moves faster from high to low and the number of opinion clusters decreases.

***Definition2***: *For all agents in a group who change their opinion*, *the ultimate opinion values are called a group consensus opinion.*

**Fig 5:**
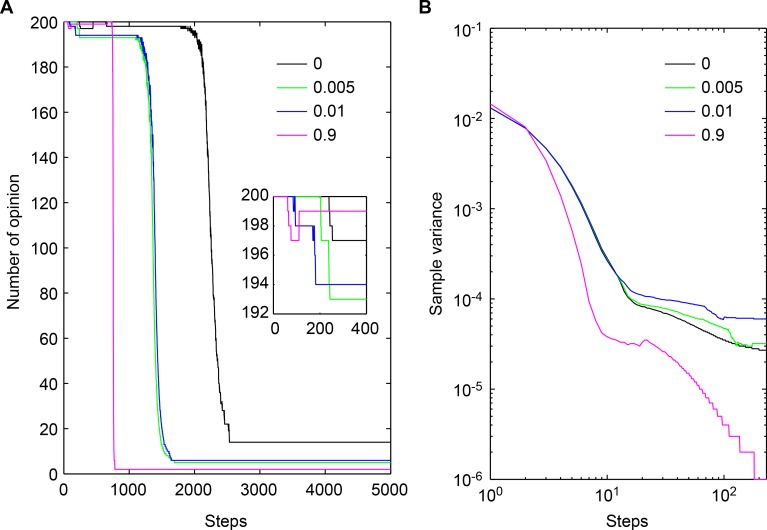
The analysis of consensus opinions with different values of Δδ. (a) The formation process of a consensus opinion; the embedded figure shows the number of opinions changing in 400 time steps. (b) The sample variance of opinions. The black, green, blue, and red lines denote Δδ = 0, Δδ = 0.005, Δδ = 0.01 and Δδ = 0.9, respectively.

To analyze how a group consensus opinion forms and the number of consensus opinions in a group, we demonstrate convergence over time in Fig 5.

The process of convergence is shown in 5,000 time steps and at the beginning of convergence. Those agents with similar opinions in the group begin to share the same opinions as their neighbors; the number of opinions are 14,5,5, and 2 in the four conditions at 5000 time step. However, in Condition I, the number 14 will be decreased in theory over longer time steps, and it is more difficult to converge to the consensus opinion in Condition I than in the other conditions. The confidence variation is the main reason to accelerate convergence, and when the confidence variation is larger, convergence is more rapid. However, there are certain phenomena that are not initially clear between Conditions II and III. In Condition II, it converges a little faster and earlier than is the case in Condition III. We explain this phenomenon in Fig 5B, the opinion sample variance, where the degree of sample variance in the Condition II is shown to be lower than in Condition III; we infer that this sample variance is the primary reason for the different convergences in Condition II.

### A special case analysis of Δδ = 1

Δδ = 1 is a special case because it only concernsthe network structure. For $\varepsilon_{i}(t)=\frac{k_{i}^{+}(t-1)}{k_{i}^{+}(t-1)+k_{i}^{-}(t-1)}$, the in/out degree of the real network is crucial for an agent’s opinion. Ifwe suppose that the in and out degrees are 1, then we have the following experimental results.

**Fig 6:**
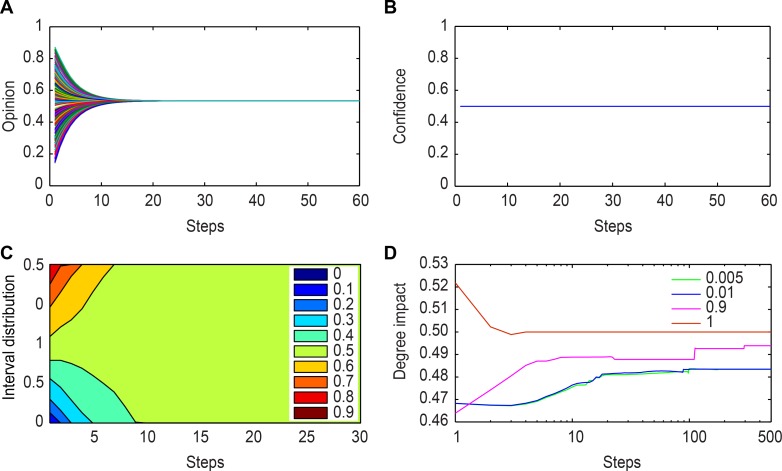
Analysis for Δδ = 1. (a)Opinion evolution. (b) Confidence evolution. (c) An area figure showing the interval distribution of opinion evolution overtime. The areas with different colors denote the opinion interval overtime. (d) The average degree impact of neighbors. The green, blue, red, and orange lines denote the impact of Δδ = 0.005, Δδ = 0.01, Δδ = 0.9, and Δδ = 1, respectively.

Fig 6A and 6B show the opinion evolution and confidence level overtime. The trust threshold value is always 0.5,all agents are neighbors at the outset, and opinion clusters and the group consensus opinion both converge to 0.5, as shown in Fig 6C. Theorem 3 shows that if $\frac{k_{i}^{+}(t-1)}{k_{i}^{+}(t-1)+k_{i}^{-}(t-1)}=\varepsilon_{i}(0)$, it will converge to the consensus opinion. Because *ε*_*i*_(0) = 0.5 at the beginning, then $\frac{k_{i}^{+}(t-1)}{k_{i}^{+}(t-1)+k_{i}^{-}(t-1)}=0.5$, and the network is connected completely which is also known form the adjacent matrixes. Fig 6D shows the influence of $\frac{k_{i}^{+}(t-1)}{k_{i}^{+}(t-1)+k_{i}^{-}(t-1)}$; when there are more increments for Δδ, the agent’s opinion has greater influence.

The real network structure plays an important role in opinion evolution, although this structure will be explored in another paper.

## Conclusion

We advance an opinion formation model with time-varying bounded confidence that is based on how people communicate in daily life; the model involves confidence variation and the degree of neighbor node. We provided the mathematical definition of the model and figures regarding opinion evolution and time-varying bounded confidence that were obtained under experimental conditions. We explored the evolutionary trend of opinion clusters by defining the neighbor relationship, and we found that opinions in groups converged exponentially into a stable neighbor relationship. The evolutionary trend of opinion clusters relates to the average opinion strength of the neighbor. If the average opinion strength is greater than zero, the opinion cluster will transform to a high interval. If the average opinion strength is less than zero, the opinion cluster will transform to a low interval. If the strength of the opinion equals zero, then neighbors share the same opinions, i.e., group consensus opinions. Moreover, the confidence variation increment is the main factor in shortening the convergence time. We explored time-varying bounded confidence using 0 < Δ*δ* ≤ 1, and found that the threshold of bounded confidence converges at a rate to consensus; meanwhile, $K=\frac{1}{M}\sum_{i}\varepsilon_{i}(0)$. Δδ = 1 is a special condition involving the real network structure. Our work is preliminary, and more in-depth research will be conducted in the future.

Confidence variation will affect the number of opinion clusters, the convergence of the trust threshold value and the degree of the opinion sample dispersion. When Δδ is larger, the stable neighbor relationships will form faster, and the effect on opinion evolution will be greater.

Opinion clusters are also related to the characteristics of individuals. Agents with a high opinion value but a low trust threshold value find it difficult to incorporate their opinion cluster into other groups, even when the confidence variation increment is sufficiently large. Notably, the role of individual characteristics in consensus will be discussed in future work.

## Supporting information

S1 Dataset Dataset for time-varying opinion formation. Dataset for Opinions and Confidence with Δδ = 0; Δδ = 0.0005; Δδ = 0.01; and Δδ = 0.9; and dataset of the instance of Agent117.
(XLSX)
